# MLBCD: a machine learning tool for big clinical data

**DOI:** 10.1186/s13755-015-0011-0

**Published:** 2015-09-28

**Authors:** Gang Luo

**Affiliations:** Department of Biomedical Informatics, University of Utah, Suite 140, 421 Wakara Way, Salt Lake City, UT 84108 USA

**Keywords:** Machine learning, Big clinical data, Automatic algorithm selection, Automatic hyper-parameter value selection, Entity–Attribute–Value, Pivot

## Abstract

**Background:**

Predictive modeling is fundamental for extracting value from large clinical data sets, or “big clinical data,” advancing clinical research, and improving healthcare. Machine learning is a powerful approach to predictive modeling. Two factors make machine learning challenging for healthcare researchers. First, before training a machine learning model, the values of one or more model parameters called hyper-parameters must typically be specified. Due to their inexperience with machine learning, it is hard for healthcare researchers to choose an appropriate algorithm and hyper-parameter values. Second, many clinical data are stored in a special format. These data must be iteratively transformed into the relational table format before conducting predictive modeling. This transformation is time-consuming and requires computing expertise.

**Methods:**

This paper presents our vision for and design of MLBCD (Machine Learning for Big Clinical Data), a new software system aiming to address these challenges and facilitate building machine learning predictive models using big clinical data.

**Results:**

The paper describes MLBCD’s design in detail.

**Conclusions:**

By making machine learning accessible to healthcare researchers, MLBCD will open the use of big clinical data and increase the ability to foster biomedical discovery and improve care.

## Background

The healthcare industry collects large amounts of clinical data from diverse sources including electronic medical records, sensors, and mobile devices. These large clinical data sets, or “big clinical data,” provide opportunities to advance clinical care and biomedical research. Predictive analytics leverage these large, heterogeneous data sets to further knowledge and foster discovery. Predictive modeling can facilitate appropriate and timely care by forecasting an individual’s health risk, clinical course, or outcome. Approaches to predictive modeling include statistical methods such as logistic regression and machine learning methods that improve automatically through experience [1], such as support vector machine, neural network, decision tree, and random forest. Compared to statistical methods, machine learning can increase prediction accuracy, sometimes doubling it, with less strict assumptions, e.g., on data distribution [2–4].

Two major aspects of machine learning require significant computing expertise and are poorly supported by existing machine learning software such as Weka [5], RapidMiner, R, and KNIME [6], making machine learning inaccessible to many healthcare researchers who use clinical data to do research [7–9]. First, before a machine learning model can be trained, an algorithm and hyper-parameter values must be chosen. An example hyper-parameter is the number of hidden layers in a neural network. The chosen algorithm and hyper-parameter values can have a large impact on the resulting model’s performance, sometimes changing accuracy from 1 to 95 % [8]. Selecting an effective algorithm and hyper-parameter values is currently an art, which requires deep machine learning knowledge as well as repeated trials. It has been widely recognized that this is beyond the ability of layman users with limited computing expertise, and also frequently a non-trivial task even for machine learning experts [7, 8, 10–12]. Emerging evidence suggests that automatic search methods for the optimal algorithm and hyper-parameter values can achieve equally good or better results than careful manual tuning by machine learning experts [10, 13]. However, when a large variety of algorithms is considered, prior efforts such as Auto-WEKA [8], hyperopt-sklearn [13], and MLbase [7, 14] cannot quickly determine the optimal algorithm and hyper-parameter values for a large data set, limiting their usefulness in practice.

A major obstacle to automatic search is that a long time is needed to examine a combination of an algorithm and hyper-parameter values on the entire data set. When determining an optimal combination, prior efforts at automation examine many combinations on the entire data set. On a data set with a modest number of data instances and attributes, such as several thousand rows and several dozen attributes, this can last several days [8]. In practical applications, search time can be hundreds or thousands of times longer for three reasons: (1) The process of conducting machine learning is iterative. If a particular set of clinical parameters yields low prediction accuracy, the analyst will probably look at other unused, available clinical parameters that may be predictive. A new search is required for each iteration. (2) A data set can consist of many data instances, e.g., from several healthcare systems. (3) A data set can include many attributes, like those extracted from textual and/or genomic data. The execution time of a machine learning algorithm typically grows at least linearly with the number of attributes and superlinearly with the number of data instances. Many predictive modeling problems must be resolved for numerous diseases and outcomes to attain personalized medicine. Search time will become a bottleneck at this point, irrespective of whether it creates an issue for a predictive modeling problem.

**Fig. 1:**
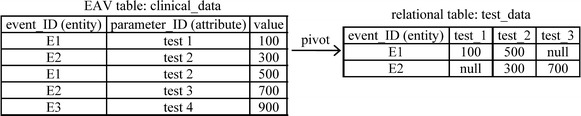
Pivot to obtain the columns for the three clinical parameters ‘test 1,’ ‘test 2,’ and ‘test 3’

The second aspect is related to the data extraction required before data analysis. Many clinical data are stored in the Entity-Attribute-Value (EAV) format (see Fig. 1) [15]. Examples of electronic medical record (EMR) systems using the EAV format include the Cerner Powerchart EMR [16], Regenstrief EMR [17], Intermountain Healthcare’s HELP EMR [18], TMR EMR [19], and Columbia-Presbyterian EMR [20]. Examples of clinical study data management systems using the EAV format include Oracle Clinical [21], Clintrial [22], TrialDB [23], i2b2 (Informatics for Integrating Biology and the Bedside), REDCap, OpenClinica, LabKey, and Opal [24, 25]. A large portion of patient-generated health data, such as those from home health equipment, in personal health records, or from mobile apps, is stored in the EAV format [26]. Even in an enterprise clinical data warehouse designed to provide data ready for analysis, some of the largest tables (e.g., the fact tables) still use the EAV format [27, 28]. In the OMOP (Observational Medical Outcomes Partnership) [29] and PCORnet (the National Patient-Centered Clinical Research Network) Common Data Models [30] and i2b2 data mart schema [31], some of the largest tables (e.g., observation, diagnosis, procedure, and lab result) use the EAV format.

The EAV data model uses tables with at least three columns: the entity, attribute, and value. Usually, the entity column identifies a clinical event and can be regarded as a patient ID and date/time stamp pair [27, page 58]. The attribute column identifies a clinical parameter. The value column contains the clinical parameter’s value. In this way, an EAV table combines many clinical parameters and their values in the attribute and value columns.

Before performing predictive modeling, EAV data must be transformed by pivot operations into relational table formats (see Fig. 1), with each clinical parameter of interest occupying its own column. Pivoting is often performed repeatedly, as machine learning is an iterative process. Since healthcare researchers with limited computing expertise are known to have difficulty writing complex database queries [32], each round of pivoting requires work from a computing professional, which creates dependencies and consumes significant time and computing resources. Traditional pivoting techniques often require repeatedly processing large clinical data sets and/or performing multiple join operations [33–35], either of which is computationally expensive.

New approaches are needed to enable healthcare researchers to build machine learning predictive models on big clinical data efficiently and independently. To fill the gap, we present in this paper the design of a new software system called MLBCD (Machine Learning for Big Clinical Data) supporting the whole process of iterative machine learning on big clinical data, including clinical parameter extraction, feature construction, machine learning algorithm and hyper-parameter selection, model building, and model evaluation. MLBCD can be used once the researcher has defined the study population and research question, has obtained the clinical data set, and has finished data preparation [36] including cleaning and filling in missing values. For clinical data, filling in missing values usually requires applying medical knowledge, and therefore is unsuitable for complete automation.

This work makes the following innovative contributions:

1. We present the first software supporting the whole process of iterative machine learning on big clinical data, from clinical parameter extraction to model evaluation. Currently no such software exists.
2. We present a new method to provide a solution to a long-standing open problem in machine learning that has been widely recognized in the literature [7, 11, 12, 14]. Our method efficiently (in less time) and automatically searches for the optimal machine learning algorithm and hyper-parameter values for a given machine learning problem. Existing automatic search methods are inefficient. Our method uses sampling to search for the optimal algorithm and hyper-parameter values concurrently. This has never been done before. Our method uses new techniques such as handling high-performance and low-performance combinations of hyper-parameter values in different ways. With proper extensions, these techniques can be used for handling other problems in stochastic optimization.
3. We present the first implementation method of efficient pivoting techniques using the MapReduce framework [37] for distributed computing. Pivot operations are essential for analyzing clinical data, but are not supported by existing big data software for distributed computing such as Hadoop [38] and Spark [39].
4. MLBCD offers new features tailored to healthcare researchers’ needs, such as the options of producing only interpretable models, specifying forced inclusion of a subset of input variables in the model, and displaying the used input variables in descending order of importance with cumulative impact on prediction accuracy. Existing machine learning software systems are not tailored to healthcare researchers’ needs.

## Methods

MLBCD integrates techniques of fast pivoting, visual query building, efficient and automatic selection of machine learning algorithms and hyper-parameter values, and scalable machine learning. It provides an intuitive graphical user interface for each step of the analytical process and can run on a cluster of commodity computers for scalable parallel processing. MLBCD uses a new method for efficiently and automatically searching for the optimal machine learning algorithm and hyper-parameter values for a given machine learning problem. MLBCD also provides the first implementation of efficient pivoting techniques using the MapReduce framework [37] for distributed computing.

After obtaining EAV data containing potentially predictive clinical parameters, MLBCD can be used to perform fast iterative machine learning. For example, hundreds of thousands of clinical parameters exist in an EMR [27, page 56]. An analyst typically starts the analytical process from a few clinical parameters such as lab tests. With the EAV tables containing all lab tests and their result values, the analyst can use MLBCD to iteratively add more lab tests for analysis until satisfactory prediction accuracy is reached.

## Results and discussion

This part of the paper is organized as follows. “Existing big data software” provides some background on existing big data software relevant to MLBCD. “The design of MLBCD” presents the design of MLBCD. “An automatic selection method for machine learning algorithms and hyper-parameter values**”** describes the efficient and automatic selection method for machine learning algorithms and hyper-parameter values used in MLBCD. “Evaluation plan” mentions our evaluation plan for MLBCD. “Related work” discusses related work.

### Existing big data software

In this section, we provide some background on existing big data software relevant to MLBCD. Modern big data software for distributed computing is developed to support large-scale data-intensive applications not handled well by parallel relational database systems. These big data software systems typically run on a cluster of commodity computers, borrow many techniques from parallel relational database systems, and provide new functions beyond those supported by parallel relational database systems.

Hadoop [38] and Spark [39] are two widely used, open source, big data software systems. Hadoop implements Google’s MapReduce framework [37] for distributed computing using the Map and Reduce functions. The Map function converts an input element into zero or more key-value pairs. The Reduce function converts a key and its list of associated values into zero or more key-value pairs that can be of another type. Data are stored in the Hadoop distributed file system, the open source implementation of Google’s BigTable file system [40]. Hadoop is unsuitable for iterative and interactive jobs, as job execution usually requires repeated reading and writing of data from and to disk, incurring significant overhead [39]. Structured Query Language (SQL) is the standard query language for relational database systems. SQL’s declarative nature allows easier programming than by low level Map and Reduce functions. Hive [41] is a software system supporting a large portion of SQL on top of Hadoop.

To overcome Hadoop’s shortcomings, Spark [39] was developed on top of the Hadoop distributed file system. To improve performance, Spark executes most operations in memory and avoids disk inputs/outputs when possible. Like Hadoop, Spark supports the MapReduce framework. Spark SQL [42, 43] is a software system supporting many relational operators, a large portion of SQL, and other functions on top of Spark. MLlib [7, 44, 45] is Spark’s machine learning library. Spark can run SQL queries at a speed comparable to parallel relational database systems and up to 100 times faster than Hive, and iterative machine learning algorithms >100 times faster than Hadoop [42]. Neither Spark SQL nor Hive supports the pivot operator. MLBCD is developed using Spark, Spark SQL, MLlib, and new techniques to address existing software’s limitations.

### The design of MLBCD

In this section, we present the design of MLBCD. During iterative machine learning on big clinical data, three sequential steps are executed repeatedly. First, a set of clinical parameters is extracted from EAV data into relational table formats by pivoting. Second, raw clinical parameters are transformed to construct features, a.k.a. input variables or independent variables, of the predictive models to be built. This step is optional and often done by executing SQL queries. If this step is omitted, raw clinical parameters will be the input variables of the predictive models to be built. Third, one or more predictive models are built on the current set of clinical parameters and evaluated. If model performance is unsatisfactory, the analyst can add more clinical parameters and restart from the first step.

**Fig. 2:**
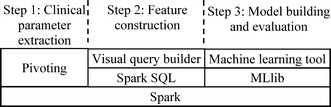
Architecture of MLBCD

MLBCD covers all three steps and supports the whole process of iterative machine learning on big clinical data. MLBCD provides a separate intuitive graphical user interface for each step. At any time, the user can move easily between the three steps at will. MLBCD uses Spark as its basis for distributed computing, allowing it to run on a single computer as well as on a cluster of commodity computers for parallel processing. As detailed below, MLBCD is built using the open source software systems Spark, Spark SQL, MLlib, and SQLeo [46], each of which either supports a Java application programming interface or is written in Java. MLBCD is written mainly in Java so it can call the functions in and interface with these software systems. Figure 2 shows MLBCD’s architecture. In the following, we describe the software component for each of the three steps.

#### Step 1: Clinical parameter extraction

In the first step, MLBCD performs pivoting on Spark to extract desired clinical parameters. The pivot operator is currently not supported by Spark SQL, nor well supported by existing large-scale relational database systems. We use the MapReduce framework [37] to support the pivot operator on Spark and implement three efficient pivoting techniques that we have developed in Luo and Frey [33]. The first technique performs early removal of EAV tuples related to unneeded clinical parameters. The second technique facilitates pivoting across several EAV tables. The third technique supports multi-query optimization.

Our techniques fit well with MapReduce by forming one key-value pair per EAV tuple in the Map function. The key is the entity column of the EAV tuple. The value is the combination of the attribute and value columns of the EAV tuple. In the Map function, EAV tuples related to unneeded clinical parameters are filtered out early on [33]. The Reduce function combines all relevant EAV tuples with the same entity value from one or more EAV tables into a relational tuple. The Reduce function can write to multiple files for each record processed [38, 41], supporting multi-query optimization.

To let users with limited computing expertise avoid writing SQL-like statements for pivoting, MLBCD provides an intuitive graphical user interface to guide users through the pivoting process. In MLBCD’s input interface, the user specifies sequentially (a) the EAV data’s storage location, such as the name of a comma-separated values (CSV) file in the local file system, a file in the Hadoop distributed file system, or an EAV table in a relational database; (b) the fields of the EAV data corresponding to the entity, attribute, and value columns, respectively; and (c) desired clinical parameters. Whenever possible, the user will input by selecting from a list or navigating a directory of items rather than typing. After the user provides inputs and clicks “Extract clinical parameters”, MLBCD automatically loads the EAV data into Spark, then extracts the specified clinical parameters into relational table formats using the pivot operator on Spark. By default, the extracted relational data are stored in Spark’s default persistent storage space, the Hadoop distributed file system. In MLBCD’s input interface, the user can optionally modify the storage location of the relational data to be extracted, e.g., if the user wants to export the relational data as a CSV file for use by other programs.

As mentioned in Luo and Frey [33], there are three possible cases of pivoting: (a) pivoting on a single EAV table to generate a relational table; (b) pivoting across several EAV tables to generate a relational table from data scattered across them; and (c) performing multiple pivot operations on the same EAV table or across the same set of EAV tables to generate multiple relational tables simultaneously. MLBCD’s input interface includes one tab for each case. After completing pivoting, MLBCD displays in its output interface the first few tuples in each relational table generated. This can help the user ensure that pivoting has been done properly.

Some clinical data such as patient demographics are stored in the relational table format. MLBCD provides an intuitive graphical user interface to allow importing relational data, e.g., from a CSV file or relational database, into Spark. Both clinical data originally stored in the EAV format and clinical data stored in the relational table format then become available for the subsequent analytical process.

#### Step 2: Feature construction

In the second step, raw clinical parameters are transformed to construct features. This will typically be done by using Spark SQL to execute SQL statements on the relational data extracted in Step 1. MLBCD provides a visual query builder to help users form SQL statements. Visual query building is widely used in relational database systems. A visual query builder provides an intuitive graphical user interface, in which users form SQL statements visually. For instance, to form a basic SQL query joining two tables, the user only needs to select the two tables through drag and drop, draw a line connecting their join attributes, and then check the attributes that will appear in the results.

A good way to write the visual query builder in MLBCD is to modify the source code of SQLeo [46], an open source visual query builder written in Java. SQLeo currently supports several relational database systems, such as Oracle and PostgreSQL, but not Spark. The modification lets SQLeo interact with Spark SQL using Java Database Connectivity (JDBC) supported by SQLeo and Spark SQL. After the visual query builder forms a SQL statement and the user clicks “Run statement”, MLBCD uses Spark SQL to execute the SQL statement.

In addition to the visual query builder, MLBCD provides a command line interface for Spark. Advanced users can use the command line interface to perform operations supported by Spark and Spark SQL.

#### Step 3: Model building and evaluation

In the third step, machine learning models are built on the current set of clinical parameters and evaluated. MLBCD integrates machine learning functions of MLlib [7, 44, 45] by modifying the source code and/or calling the Java application programming interface of MLlib. Recall that MLlib is Spark’s distributed machine learning library and can run on a cluster of computers for parallel processing. MLlib implements multiple machine learning algorithms and feature selection techniques, all of which are supported by MLBCD.

Like Weka [5], MLBCD provides an intuitive graphical user interface for machine learning. Weka is the most widely used open source machine learning and data mining toolkit. Weka’s graphical user interface supports feature selection (optional), model building, and model evaluation. In the input interface, the user specifies the data file, independent variables, dependent variable, machine learning algorithm, and its hyper-parameter values. After the user clicks “Start,” Weka builds the model and presents its performance metrics. MLBCD’s graphical user interface for machine learning works similarly with one major difference. In Weka’s input interface, the user must select an algorithm before building the model. This requires computing expertise. Like Auto-WEKA [8], MLBCD treats the choice of feature selection technique as a hyper-parameter and uses the method described in “An automatic selection method for machine learning algorithms and hyper-parameter values” to automatically search for the optimal algorithm, feature selection technique, and hyper-parameter values. If desired, the user can make changes in MLBCD’s input interface. If the resulting model’s accuracy is lower than a pre-determined threshold, such as area under the receiver operating characteristic curve (AUC) <0.8 [47, page 177], MLBCD automatically prompts the user to consider returning to Step 1 to add additional clinical parameters.

By default, MLBCD considers all input variables, machine learning algorithms, and feature selection techniques. In the input interface, the user can optionally specify a subset of input variables deemed important based on medical knowledge and must be included in the model. In this case, feature selection will be applied only to the other input variables. The user can also optionally specify the feature selection techniques and/or algorithms to be explored, possibly based on a desired property. For instance, the user may want interpretable models such as decision tree and *k*-nearest neighbor (similar patients) [48]. In the output interface, a receiver operating characteristic (ROC) curve is displayed for binary classification. The user can mouse over the ROC curve to exploit trade-offs between sensitivity and specificity. To help simplify the model, the user can opt to see the used input variables sorted in descending order of importance, e.g., using backward feature elimination [1]. For each input variable, the accuracy of the model using all input variables up to it is shown. Often, not every clinical parameter used in the model is routinely collected in all healthcare systems’ databases. By determining the set of clinical parameters essential for high accuracy, the user can simplify the model so other healthcare systems are more likely to adopt it.

### An automatic selection method for machine learning algorithms and hyper-parameter values

In this section, we present a new method for efficiently and automatically searching for the optimal algorithm and hyper-parameter values for a given machine learning problem. MLBCD uses this method to address existing automatic search methods’ inefficiencies mentioned in the Introduction. Our discussion focuses on a large data set. With some modifications, the new method will also apply to relatively small data sets, e.g., by using the test results on a few random combinations of hyper-parameter values to quickly determine whether a machine learning algorithm should be eliminated from further consideration. Any given accuracy measure, such as AUC, can be used in our method.

In “Overview of the automatic search method”, we give an overview of the new automatic search method. In “Background on hyper-parameters”, we provide some background on hyper-parameters. In “Review of the sequential model-based optimization method”, we briefly review the sequential model-based optimization method. We describe the observations and insights based on which the new automatic search method is designed in “Observations and insights”. In “The training and test samples**”**–“The iterative search process**”**, we present various parts of the new automatic search method in detail.

#### Overview of the automatic search method

We consider all machine learning algorithms applicable to the data set. We focus on the common case that no experimental results on previous machine learning problems are available. If this is not the case, experimental results on previous machine learning problems can be used to help select a good starting point of the search process for the current machine learning problem, e.g., in a way similar to that in Feurer et al. [49], and improve search efficiency.

**Fig. 3:**
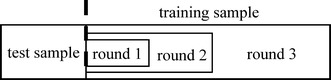
An illustration of progressive sampling used in our automatic search method

The entire space of machine learning algorithms and hyper-parameter values is extremely large due to the large number of algorithms and possible hyper-parameter values. To efficiently and automatically search for the optimal algorithm and hyper-parameter values, we perform progressive sampling, filtering, and fine-tuning to quickly narrow down the search space. As shown in Fig. 3, our key idea is to use progressive sampling to generate a sequence of random samples of the data set, one nested within another [50]. Inexpensive tests are conducted on small samples of the data set to eliminate unpromising algorithms and identify unpromising combinations of hyper-parameter values as early and as much as possible. More computational resources are devoted to fine-tuning promising algorithms and combinations of hyper-parameter values on larger samples of the data set. The search process is repeated for one or more rounds. As the sample of the data set expands, the search space shrinks. In the last round, (a large part of) the entire data set is used to find an optimal combination of an algorithm and hyper-parameter values. Sampling has been used before for searching for the optimal machine learning algorithm [9, 50–57], but not for searching for the optimal algorithm and hyper-parameter values concurrently.

#### Background on hyper-parameters

**Fig. 4:**
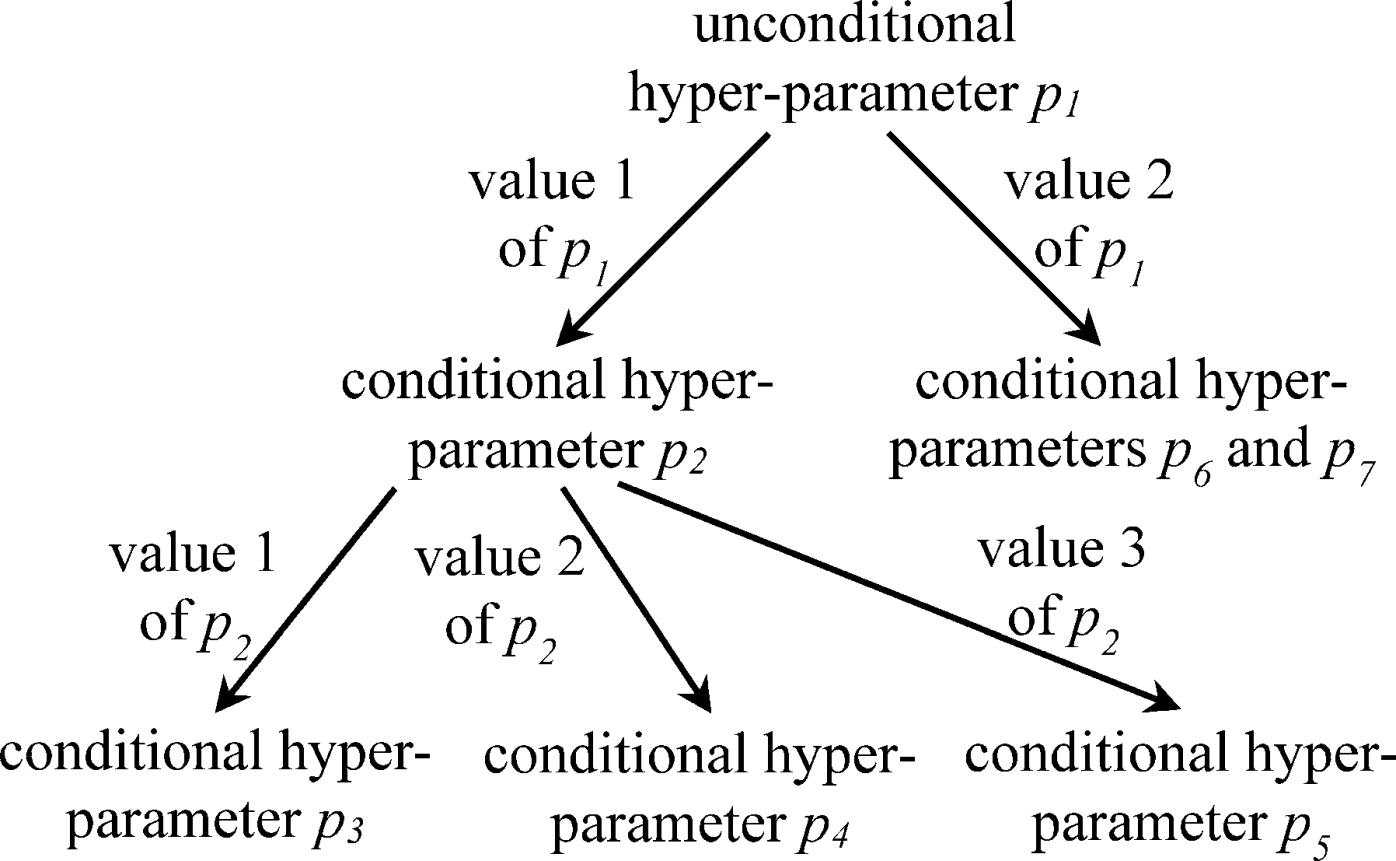
An example dependency graph formed by all hyper-parameters of a machine learning algorithm

In this section, we provide some background on hyper-parameters needed for describing our automatic search method. There are two types of hyper-parameters: conditional and unconditional. An unconditional hyper-parameter is always used. In contrast, the relevance of a conditional hyper-parameter depends on the value of another hyper-parameter. For instance, for neural network, the hyper-parameter of the number of hidden units in the second layer is relevant only if the hyper-parameter of the number of layers in the neural network is ≥2. As shown in Fig. 4, all hyper-parameters of a machine learning algorithm form a tree or directed acyclic graph.

#### Review of the sequential model-based optimization method

Our goal is to automatically search for the optimal machine learning algorithm and hyper-parameter values. The current approach for handling this problem [8, 13] is to treat the choice of algorithm as a new hyper-parameter at the root level and map this problem to the problem of searching for the optimal hyper-parameter values. Sequential model-based optimization [8, 10, 58, 59], also known as Bayesian optimization, is the state-of-the-art method for conducting this search. It proceeds in rounds. In each round, a new combination of hyper-parameter values is selected for testing.

More specifically, the sequential model-based optimization method first builds a regression model to predict a machine learning model’s accuracy based on hyper-parameter values. Random forest is a commonly used regression modeling approach [8] and has been shown to outperform several other approaches for making this prediction [60]. For any combination of hyper-parameter values, evaluating the regression model’s output is cheaper than training the machine learning model and evaluating its accuracy on the data set. When training the regression model and using it to make predictions, inactive conditional hyper-parameters are set to their default values [8].

Next, the following three steps are iterated until a pre-determined stopping criterion is satisfied: use the regression model to identify a promising combination of hyper-parameter values *c* to evaluate next; train a machine learning model and evaluate its accuracy *a* on the data set at *c*; and use the new data point (*c*, *a*) to update the regression model. In practice, it is possible for the regression model to be misdirected. To achieve robust performance even if this situation occurs, every second combination of hyper-parameter values to evaluate next is chosen at random. In this way, new areas of the hyper-parameter space can be explored [8].

#### Observations and insights

Our automatic search method is designed based on the following observations and insights.

##### Insight 1

It is time-consuming to test a combination of a machine learning algorithm and hyper-parameter values on the whole data set. It is much faster to test this combination on a (relatively) small random sample of the data set.

##### Insight 2

**Fig. 5:**
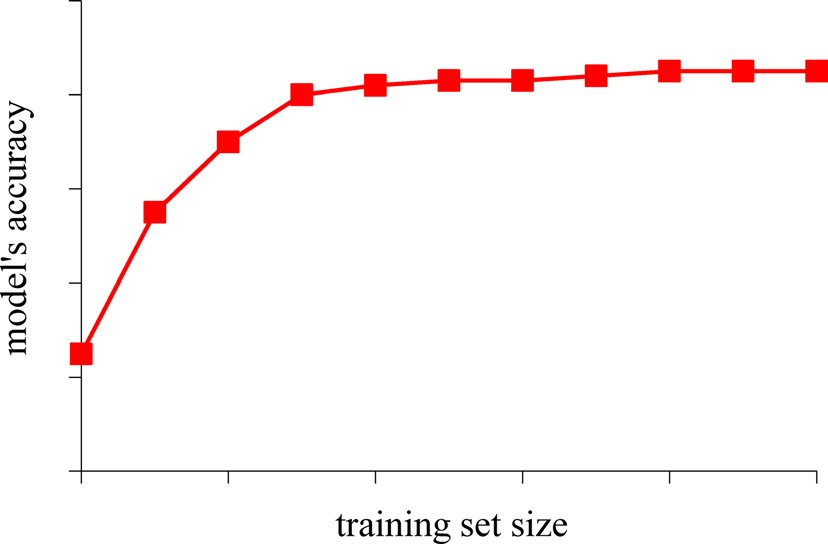
Training set size vs. model’s accuracy

As shown in Fig. 5, for a specific combination of a machine learning algorithm and hyper-parameter values, the model’s accuracy usually increases with a larger training set. When the training set becomes large enough, the model’s accuracy will stop increasing (much) [50]. A random sample of the data set can be used to train the model and estimate its accuracy. As long as the sample is not too small, the estimate will give a rough idea of the accuracy that can be achieved when (a large part of) the whole data set is used to train the model.

##### Insight 3

Consider two machine learning algorithms. As shown in Petrak [9], if one algorithm significantly outperforms another in accuracy when a not-too-small, random sample of the data set is used to train the model, the former is also likely to outperform the latter in accuracy when (a large part of) the whole data set is used to train the model.

##### Insight 4

Consider a specific machine learning algorithm and data set. To find out the highest accuracy the algorithm can possibly achieve on the data set, it is insufficient to test only one combination of hyper-parameter values. As shown in Bergstra and Bengio [61], random search is an effective approach for searching the space of all possible combinations of hyper-parameter values. We can test a few random combinations and find the highest accuracy they achieve on the data set. This accuracy will give a rough idea of the highest accuracy the algorithm can possibly achieve on the data set with optimal hyper-parameter values.

##### Insight 5

The per data instance overhead of training a model is usually much higher than that of testing the model. In other words, training a model on a given number of data instances takes much longer than testing a model on these data instances. Moreover, due to repeated training, multi-fold cross validation is time-consuming to perform. Consider a specific data set and combination of a machine learning algorithm and hyper-parameter values. For quickly obtaining a rough and relatively robust estimate of the accuracy that the combination can achieve on the data set, it would be good to train the model on one sample of the data set and test the model on another disjoint, relatively large sample of the data set. This would be more efficient than performing multi-fold cross validation on the first sample of the data set [9].

##### Insight 6

In practice, there are often multiple good combinations of machine learning algorithms and hyper-parameter values, each of which can achieve accuracy close to that of an absolutely optimal combination. Our goal is not to find the absolutely optimal combination and build the absolutely optimal model. Thus, an exhaustive search of all possible combinations is unnecessary. Instead, our goal is to find a good combination and build a good model whose accuracy is close to that of an absolutely optimal model in a reasonably short amount of time. This is particularly important for performing fast iterative analytics on big clinical data. For instance, knowing that a good combination can achieve only a low level of accuracy can trigger consideration of feature engineering and/or other alternatives. Then no time needs to be spent on continuing searching for a much better combination that is unlikely to exist. If time permits, further fine-tuning of the best combination and/or model found so far can be conducted in the background, like the way MLbase works [7].

##### Insight 7

There are three types of machine learning algorithms: base, meta, and ensemble [8]. A base algorithm such as naive Bayes can be used independently. A meta algorithm such as bagging takes a base algorithm together with its hyper-parameter values as an input. An ensemble algorithm such as voting takes several base algorithms as input. If a base algorithm achieves low accuracy, a meta or ensemble algorithm using it is unlikely to achieve high accuracy, at least in comparison to one using a well-performing base algorithm. In comparison, if a base algorithm achieves reasonable accuracy, a meta or ensemble algorithm using it may achieve high accuracy, regardless of whether it is the best performing base algorithm. This insight can be used to improve search efficiency.

Due to its inherent complexity, a meta or ensemble algorithm is more expensive to test than a base algorithm used by it. In the first few rounds of the search process, we test base algorithms, but not meta or ensemble algorithms, on relatively small samples of the data set. Unless a base algorithm looks promising, we will not proceed to test the meta or ensemble algorithms using it in later rounds of the search process. In other words, poorly performing base algorithms are eliminated in the first few rounds. The subsequent rounds focus on testing the remaining base algorithms that perform reasonably well, as well as the meta and ensemble algorithms using them. In this way, some unnecessary tests of meta or ensemble algorithms are saved.

##### Insight 8

As mentioned in “ Review of the sequential model-based optimization method“, in searching for the optimal machine learning algorithm and hyper-parameter values, the current approach [8, 13] treats the choice of algorithm as a new hyper-parameter at the root level and handles it in the same way as the other hyper-parameters, which we call *regular hyper*-*parameters*. This approach is suboptimal because the new hyper-parameter has different properties than regular hyper-parameters.

The choice of machine learning algorithm tends to affect the resulting model’s accuracy much more than that of a regular hyper-parameter’s value. For a specific machine learning problem and algorithm, usually only a few hyper-parameters of the algorithm matter much, while the others have little impact on the model’s accuracy [61]. Also, if a small change is made to a numerical hyper-parameter’s value, the model’s accuracy will typically vary only slightly. In contrast, once the algorithm changes, the model’s accuracy will often be greatly altered.

The choice of machine learning algorithm affects the relevance of many more hyper-parameters than a regular hyper-parameter. Once an algorithm is selected, most hyper-parameters of the other algorithms, i.e., most regular hyper-parameters, become irrelevant. In contrast, within the context of a specific algorithm, the value of a regular hyper-parameter affects the relevance of few other hyper-parameters or none at all.

By handling the choice of machine learning algorithm and regular hyper-parameters in somewhat different ways, the above two properties can be used to improve search efficiency. For example, to guide the search direction, a regression model is often built to predict a machine learning model’s accuracy based on hyper-parameter values [8, 10, 58, 59]. Instead of building a single aggregate regression model for all hyper-parameters and algorithms, we can build a separate regression model for each algorithm and its hyper-parameters. Due to significantly reduced dimensionality, the regression models for individual algorithms can be made more accurate than the aggregate one for all algorithms within the same resource constraints. As another example, by eliminating unpromising algorithms in the first few rounds of the search process, these algorithms’ hyper-parameters no longer need to be considered further. Then in subsequent rounds of the search process, we can focus on fine-tuning the remaining promising algorithms’ hyper-parameter values. The reduced search space makes it easier to find good search results.

##### Insight 9

In the sequential model-based optimization method, a regression model is used to select a new combination of hyper-parameter values for testing in each round. The new combination should be likely to achieve high accuracy. The regression model is built using accuracy estimates for the combinations of hyper-parameter values that have been tested previously.

The combinations of hyper-parameter values can be classified into two types: the ones achieving high accuracy (high-performance) and the ones achieving low accuracy (low-performance). As mentioned in Eggensperger et al. [60], which new combination is selected for testing in each round tends to be impacted mainly by the accuracy estimates for the high-performance combinations. The low-performance combinations are mainly used to indicate low-performance regions in the search space that should be avoided. Hence, it is more important to obtain precise accuracy estimates for the high-performance combinations than for the low-performance ones. If a precise accuracy estimate is too expensive to obtain for a low-performance combination, we can try to quickly obtain a rough accuracy estimate for it. As long as the rough accuracy estimate indicates that the combination has low performance, it is often good enough for selecting a good new combination for testing in each round.

##### Details of the automatic search method

In the following, we present the details of our automatic search method. We proceed in multiple rounds and use progressive sampling to quickly narrow down the search space. In each round, we use an accuracy difference threshold *τ* and two disjoint, random samples of the data set: one termed the *training sample* and another termed the *test sample*. The training sample is used to train models. The test sample is used to evaluate each trained model’s accuracy. The accuracy reflects how promising the model’s corresponding machine learning algorithm and hyper-parameter values look by the current round.

#### The training and test samples

As shown in Fig. 3, the training sample expands from one round to the next. An effective expansion method is to increase the training sample size exponentially, e.g., double the training sample size each round [50].

The initial training sample’s size needs to fulfill two requirements. First, it should be large enough to give a rough idea of the accuracy that can be achieved when (a large part of) the whole data set is used to train a model. Second, it should not be too large to make model training too slow. Otherwise, we cannot quickly eliminate unpromising machine learning algorithms and identify unpromising combinations of hyper-parameter values.

One approach fulfilling both requirements is to set the initial training sample’s size to the maximum of the following two values: (1) a pre-determined constant such as 1000 and (2) the number of input variables (a.k.a. independent variables) of the model multiplied by another pre-determined constant, such as 10. By comparison, existing work on using sampling to search for the optimal machine learning algorithm typically uses a fixed sample size as the starting point [9, 50–57]. In the presence of many input variables, this fixed size may be too small, leading to highly inaccurate estimates of the potential of an algorithm and/or combination of hyper-parameter values and to misguidance of the subsequent search process.

The test sample remains the same over rounds, e.g., with a fixed size of 3000. The test sample needs to be large enough to give a relatively robust estimate of the model’s accuracy, but not necessarily more than that. In fact, to avoid spending an excessive amount of time testing models, the test sample should not be too large.

To efficiently and repeatedly generate random samples of the data set over rounds, the following approach is used. A random number is appended as an additional attribute to every data instance in the data set. All data instances are sorted in ascending order of the attribute. The attribute is removed during the last phase of sorting, as it is no longer needed after that. Let *n*_*training*_ and *n*_*test*_ denote the training sample size and test sample size, respectively. The first *n*_*test*_ data instances in the sorted list form the test sample. The subsequent *n*_*training*_ data instances form the training sample.

### The accuracy difference threshold

The accuracy difference threshold *τ* is used to eliminate unpromising machine learning algorithms and identify unpromising combinations of hyper-parameter values. Initially, when the training sample is relatively small, we are not quite sure of the potential of an algorithm and/or combination of hyper-parameter values. The potential is reflected by the accuracy achieved when (a large part of) the whole data set is used to train the model. To reduce the likelihood of incorrectly eliminating unpromising algorithms and identifying unpromising combinations of hyper-parameter values, *τ* should be reasonably large, such as 0.4. As the training sample expands over rounds, we will have an increasingly better idea of the potential of an algorithm and/or combination of hyper-parameter values. To use this property to expedite the process of narrowing down the search space, *τ* is decreased over rounds. One approach is to perform linear decrease, such as by 0.07 per round, until *τ* reaches a pre-determined minimum value, such as 0.05.

Using one accuracy difference threshold per round is one possible approach. Another possible approach is to use two accuracy difference thresholds per round, one for eliminating unpromising machine learning algorithms and another for identifying unpromising combinations of hyper-parameter values. The rationale for the second approach is that the accuracy difference across different algorithms may be larger than that across different combinations of hyper-parameter values for the same algorithm. Accordingly, the accuracy difference threshold for eliminating unpromising machine learning algorithms may be larger than that for identifying unpromising combinations of hyper-parameter values. It remains to be seen whether the first approach suffices, or the second approach is needed for quickly narrowing down the search space.

#### The iterative search process

The search process is done in multiple rounds. We gradually shrink the search space by eliminating unpromising machine learning algorithms and identifying unpromising combinations of hyper-parameter values over rounds. Once an algorithm is eliminated, it will no longer be used by itself in any subsequent round of the search process. In contrast, once a combination of hyper-parameter values is identified as unpromising, it will no longer be used to train the machine learning model in any subsequent round of the search process. Nevertheless, it will still be used to build regression models, which predict a machine learning model’s accuracy based on hyper-parameter values and are used to guide the search direction.

##### The first round

In the first round, we start from a relatively small training sample. The goal is to quickly eliminate machine learning algorithms that obviously look unpromising. We test every applicable algorithm. As mentioned in Smith-Miles [62], for support vector machine, using a different type of kernel essentially changes the algorithm. Hence, the use of each type of kernel would be regarded as a separate algorithm [13].

For each machine learning algorithm, we test both the combination of its default hyper-parameter values and a pre-determined number (e.g., 10) of random combinations of hyper-parameter values, if any. The combination of default hyper-parameter values, such as that in Weka [5], was usually preselected by machine learning experts to be one that performs well on various machine learning problems on average. It is a reasonably good starting point of the search process. Using it can help quickly find promising regions in the search space [49].

Consider a specific machine learning algorithm. For each combination of hyper-parameter values chosen for testing, we use the algorithm, hyper-parameter values, and training sample to train a model and estimate the model’s accuracy on the test sample. The estimated accuracy reflects, within the algorithm’s context, how promising the combination of hyper-parameter values looks by the current round. The combinations outperformed by the best one by a margin ≥*τ* in accuracy are regarded as unpromising, as none of the former is likely to outperform the latter in accuracy when (a large part of) the whole data set is used to train the model. Recall that *τ* is the accuracy difference threshold.

Across all combinations of hyper-parameter values that have been tested so far for a machine learning algorithm, the highest accuracy achieved on the test sample reflects how promising the algorithm looks by the current round. For a reason similar to the one mentioned above, we regard the algorithms outperformed by the best one by a margin ≥*τ* in accuracy as unpromising and eliminate them.

##### A subsequent round that is not the final one

In every subsequent round except for the final one, the machine learning algorithms remaining from the previous round and combinations of hyper-parameter values that look promising in the previous round serve as the basis of a reduced search space. We expand the training sample, decrease the accuracy difference threshold *τ*, and perform further filtering and fine-tuning of algorithms and combinations of hyper-parameter values. We use the training sample to obtain a more precise estimate of the potential of each pair of a remaining algorithm and a combination of hyper-parameter values that looks promising in the previous round. We also test new combinations of hyper-parameter values for the remaining algorithms.

More specifically, the following three steps are performed. In the first step, for each pair of a remaining machine learning algorithm and a combination of hyper-parameter values that looks promising in the previous round, we use the algorithm, hyper-parameter values, and training sample to train a model and estimate the model’s accuracy on the test sample. Compared to the accuracy estimate *E*_*1*_ obtained for the pair in the previous round, this accuracy estimate *E*_*2*_ is a more precise estimate of the potential of the algorithm and combination of hyper-parameter values. As a result of expansion of the training sample, we usually have *E*_*2*_ ≥ *E*_*1*_. The accuracy ratio $r = E_{2} /E_{1}$ reflects the degree of increase in accuracy.

In the second step, we select and test new combinations of hyper-parameter values, if any, for the remaining machine learning algorithms. Exploration of new areas of the search space is performed using the sequential model-based optimization method [8]. As reviewed in “Review of the sequential model-based optimization method“, this method first builds a regression model to predict a machine learning model’s accuracy based on hyper-parameter values, and then uses the regression model to select new combinations of hyper-parameter values for testing.

Traditionally, sequential model-based optimization [8, 10, 58, 59] was performed using a fixed training set. In our case, the training sample expands over rounds, affecting the machine learning model’s accuracy. We modify the sequential model-based optimization method used in Auto-WEKA [8, 58] to consider this factor. Auto-WEKA uses random forest as the regression model.

For each remaining machine learning algorithm, a separate regression model is built on its hyper-parameters, as explained in “Insight 8”. As the accuracy difference threshold *τ* is reduced, the number of still promising combinations of hyper-parameter values for the algorithm tends to decrease over rounds. If the regression model is built using only the still promising combinations of hyper-parameter values, it will have low prediction accuracy due to insufficient training data and misdirect the subsequent search process.

To address this issue, the regression model is built using all combinations of hyper-parameter values that have been tested for the machine learning algorithm so far. For a combination of hyper-parameter values *c*_*u*_ that has been regarded as unpromising by the previous round, we do not have an accuracy estimate *E*_*2*_ for it from the current training sample, because obtaining this estimate is expensive and not worthwhile. Nevertheless, we do have an accuracy estimate *E*_*1*_ for *c*_*u*_ from the previous round. For all combinations of hyper-parameter values of the algorithm that look promising in the previous round, their average accuracy ratio *avg_r* reflects the average degree of increase in accuracy due to expansion of the training sample. We multiply *E*_*1*_ by *avg_r* to obtain a rough accuracy estimate for *c*_*u*_ for the current round. As explained in “Insight 9”, this rough accuracy estimate is imprecise, but often good enough for selecting good new combinations of hyper-parameter values for testing.

Once the regression model is built for the machine learning algorithm, the following three steps are repeated for a pre-determined number of times (e.g., 8): use the regression model to identify a promising combination of hyper-parameter values *c* to evaluate next; use the training sample to train a machine learning model and evaluate its accuracy *a* on the test sample at *c*; and use the new data point (*c*, *a*) to update the regression model. To explore new areas of the hyper-parameter space, every second combination of hyper-parameter values to evaluate next is chosen at random.

In the third step, we proceed in a way similar to that in the first round of the search process to eliminate unpromising machine learning algorithms and identify unpromising combinations of hyper-parameter values.

##### Iterations of the search process

We repeat the above process for a pre-determined number of rounds (e.g., 5) until the accuracy difference threshold *τ* reaches a pre-determined minimum value, such as 0.05. As the training sample expands, the number of promising machine learning algorithms and the total number of promising combinations of hyper-parameter values tend to decrease. That is, the search space shrinks. After *τ* reaches the pre-determined minimum value, each pair of a remaining promising algorithm and a combination of hyper-parameter values has similar potential. The pair achieving the highest accuracy is the best one found.

##### The final round

In the final round, we use the whole data set and best combination of the machine learning algorithm and hyper-parameter values found to train and evaluate a model. This model is the final one returned by our automatic search method. Alternatively, we can progressively expand the training sample, use the best combination and training sample to train a model, and evaluate its accuracy on the test sample for one or more times. We stop once we have enough confidence in convergence [50], i.e., the accuracy achieved by the best combination no longer improves (much) as the training sample expands. Early stopping expedites the search process.

##### Additional details on handling different types of machine learning algorithms

As mentioned in “Insight 7”, in the first few (e.g., 4) pre-determined rounds of the search process, we test base algorithms, but not meta or ensemble algorithms. In later rounds, we test the remaining base algorithms as well as meta and ensemble algorithms using them. In each such round, base algorithms are tested before meta and ensemble algorithms.

All hyper-parameters of a meta or ensemble algorithm using one or more base algorithms can be classified into three types: the ones specifying the selections of base algorithms, the ones controlling the process of combining base algorithms, and the base algorithms’ hyper-parameters. In conducting sequential model-based optimization for the meta or ensemble algorithm, a regression model is built on the first two types of hyper-parameters. When testing the meta or ensemble algorithm, the hyper-parameters of the third type are set to the best values found for the base algorithms so far. In the first round of the search process encountering the meta or ensemble algorithm, for the first two types of hyper-parameters, we test both the combination of the algorithm’s default hyper-parameter values and a pre-determined number (e.g., 10) of random combinations of hyper-parameter values, if any. Starting from this round, if a base algorithm is eliminated, it will no longer be used by itself in any subsequent round of the search process. Nevertheless, it can still be used by a meta or ensemble algorithm in future rounds.

### Evaluation plan

MLBCD is a large software system. It will take us several years to fully implement MLBCD. In this section, we present our evaluation plan for MLBCD. Our evaluation will use a test case and be completed in three stages. During the process of building MLBCD, we will assess user needs, preferences, and requirements (Stage 1). After MLBCD is built, we will evaluate its usability among healthcare researchers (Stage 2), then its utility among both healthcare researchers and computer scientists (Stage 3).

#### Demonstration test case: overview

MLBCD will be useful for any disease. As a demonstration test case, we will use MLBCD to build new models to accurately predict asthma diagnoses in children with clinically significant bronchiolitis. Both bronchiolitis and asthma are lung diseases caused by airway inflammation. Of pediatric chronic diseases, asthma is the most common [63, 64]. Asthma affects 7.1 million children (9.6 %) in the US [65, 66], incurring an annual total direct healthcare cost of about 9.3 billion dollars [63]. Asthma is the most frequent reason for preventable pediatric hospitalization [67] and school absenteeism due to chronic conditions [68]. Bronchiolitis, a disease mostly of children under age two, is highly associated with asthma. Clinically significant bronchiolitis during infancy, defined as bronchiolitis incurring an outpatient clinic visit, emergency department visit, and/or hospitalization, precedes 31 % of cases of asthma between ages 4 and 5.5 [69]. More than 1/3 of children by age two have experienced clinically significant bronchiolitis [70], with 14–40 % eventually diagnosed with asthma [71, 72]. Clinically significant bronchiolitis increases a child’s risk of asthma 2-10 times [69, 71, 73–79]. Thus, accuracy for predicting asthma diagnoses will be higher on children with clinically significant bronchiolitis than on all children [80, 81].

In 18–75 % of asthmatic children, asthma is under-diagnosed [82–86]. Also, clinicians experience difficulty diagnosing asthma in young children [87–89]. Predictive models for asthma diagnoses can assist clinicians to make timely asthma diagnoses and start asthma treatment earlier [90], as well as help study efficacy of preventive interventions for asthma in randomized clinical trials [91, 92]. At present, >20 models for predicting asthma diagnoses in children exist, but none was accurate or built specifically for children with clinically significant bronchiolitis [80].

#### Stage 1: Assess user needs, preferences, and requirements

To create an effective and usable user interface during the process of building MLBCD, we will conduct iterative focus group sessions with 6–8 healthcare researchers to assess user needs, preferences, and requirements and develop and refine “mock” prototypes until no new needs are observed. We expect 2–4 iterations to reach saturation.

##### Subject recruitment

Through personal contact and announcement in our institute’s email lists, volunteer healthcare researchers will be recruited from the University of Utah Health Sciences Center. We will recruit 6–8 healthcare researchers with limited computing expertise and obtain informed consent before the focus groups. 6–8 participants are often considered an ideal size of a focus group [93]. Purposeful sampling will be used to maximize variation to adequately capture differences in user perspectives [94, 95]. Participants will receive pseudonyms used to link their responses to questions to protect privacy. If any healthcare researcher drops out during the study, we will recruit another one for replacement.

##### Data collection

Each focus group session will be held in a meeting room at the University of Utah Health Sciences Center and last around 1 hour. Information will be solicited through a combination of semi-structured and open-ended questions on user needs, preferences, and requirements for MLBCD’s interface. We will take notes and record the sessions as digital audio files using a laptop equipped with a microphone and the Morae^@^ usability software [96]. Use of the equipment will be clearly disclosed. In the first session, we will present the background on developing MLBCD, the purpose of the focus group, and the test case described in “Stage 2: Evaluate MLBCD’s usability among healthcare researchers”. The healthcare researchers can opt to replace the test case with any case familiar to them and will provide comments on how MLBCD’s interface should look. After the session, we will create interface mock-ups of MLBCD on paper. In each subsequent session, the healthcare researchers will be provided with the latest version of the mock-ups and asked to: (1) answer targeted questions regarding their interpretations of icons, messages, labels, and other symbols; (2) explain how they will use MLBCD to perform analytics for the test case; (3) provide comments on how the mock-ups should be modified. After each session, the focus group data will be analyzed using standard methods [97–100]. The digital audio recordings and session notes will be examined. Findings will be flagged and annotated using the Morae^@^ usability software and coded in a way similar to that described in “User feedback”. Then adjustments will be made to the mock-ups. The iterative process will continue until no new changes are identified.

We will develop a detailed user manual for MLBCD. After MLBCD is built, we will evaluate its usability and utility.

#### Stage 2: Evaluate MLBCD’s usability among healthcare researchers

Following iterative prototyping recommended by usability experts [101, 102], we will evaluate MLBCD’s usability among healthcare researchers in two rounds. In the first round, we will identify initial issues and refine MLBCD. In the second round, we will identify remaining issues and finalize MLBCD. MLBCD will apply to all diseases. As a test case, each healthcare researcher will use MLBCD to build new models to predict asthma diagnoses in children with clinically significant bronchiolitis.

##### Subject recruitment

Using the same method described in “Stage 1: Assess user needs, preferences, and requirements”, we will recruit two rounds of five healthcare researchers who are not involved in the Stage 1 study, have limited computing expertise, and are familiar with pediatric asthma and bronchiolitis. Five users are usually enough to find most usability issues [103]. Purposeful sampling will be used to ensure adequate variability. All test participants will be current on information security and privacy policy training approved by the University of Utah. After providing consent, each will be given a copy of MLBCD’s user manual and a metadata document detailing tables and columns containing attributes to be used for the evaluation study. The work will be done non-continuously, as it takes time, e.g., to extract clinical parameters.

##### Demonstration test case: details

We will use the same patient population, data set, and computing environment for both the Stage 2 and Stage 3 studies:

1. *Patient population* Our study cohort includes children who had healthcare visits (outpatient clinic visit, emergency department visit, and hospitalization) at Intermountain Healthcare facilities for bronchiolitis (ICD-9-CM discharge diagnosis code 466.1 [104] ) before age two in the past 18 years, about 97,000 unique patients. Intermountain Healthcare is the largest healthcare system in Utah, with 22 hospitals and 185 clinics.
2. *Data set* We will use a large clinical and administrative data set in the Intermountain Healthcare enterprise data warehouse. Secondary analysis will be performed on a de-identified version of the data stored on a password-protected and encrypted computer cluster. The data set includes ~400 attributes and represents electronic documentation of ~85 % of pediatric care delivered in Utah [105]. For the last 18 years, data captured cover more than 3000 patients under age two and 3700 healthcare visits at Intermountain Healthcare facilities for bronchiolitis per year. Intermountain Healthcare dedicates extensive resources to ensure data integrity and accuracy.
3. *Computing environment* All experiments will be conducted on the HIPAA-compliant Homer computer cluster at the University of Utah [106]. With proper authorization, all research team members and test participants at the University of Utah can use their university computers to access this cluster. Our analysis results will provide a cornerstone to expand testing of MLBCD on other test cases and clinical data sets in the future.

##### Information about the predictive models

Clinical and administrative attributes will be used to build machine learning models.

*Defining the prediction target* (i.e., the dependent variable): The method described in Schatz et al. [107–109] will be used to identify asthma. A patient is considered to have asthma if he/she has (1) at least one ICD-9 diagnosis code of asthma (493.xx) or (2) ≥2 “asthma-related medication dispensings (excluding oral steroids) in a 1-year period,” “including β-agonists (excluding oral terbutaline), inhaled steroids, other inhaled anti-inflammatory drugs, and oral leukotriene modifiers” [107]. Identifying asthma needs medication order and refill information. Our data set includes this information, as Intermountain Healthcare has its own health insurance plan (SelectHealth). If the Intermountain Healthcare enterprise data warehouse is missing too much refill information, we will use claim data in the all-payer claims database [110] to compensate.

A child who will ever develop asthma can benefit from timely asthma diagnosis and preventive interventions for asthma [111]. Hence, our prediction target will be ever developing asthma by a certain age. No consensus exists on the optimal cut-off age [112]. To help select an appropriate cut-off age, we will plot the cumulative rate of ever developing asthma vs. age [113–115]. The age at which the cumulative rate starts to level off can be an appropriate cut-off point, as it ensures including most children who will ever develop asthma.

Let *C* denote the selected cut-off age. For a healthcare visit for bronchiolitis that occurred in year 1, data from year 1 up to year *C* + 1 are needed for computing the dependent variable’s value. Hence, given our 18 years of data on pediatric patient encounters, we can use the first 18-*C* years of data on healthcare visits for bronchiolitis and ensure that all values of the dependent variable are computable. That is, we have 18-*C* years of effective data. If the cumulative rate of ever developing asthma does not level off, we will choose *C* = 14 to ensure that at least four years of effective data are available.

*Performance evaluation* We will use the first 16-*C* years’ effective data to train predictive models. The (17-*C*)-th and (18-*C*)-th years’ effective data will be used as the test data to obtain a model’s final accuracy estimate, reflecting use in practice. If a child incurred healthcare visits for bronchiolitis in both the training and test data, we will remove the child from the test data, as correct prediction can be made by memorizing the child’s outcome. For a similar reason, if standard, stratified tenfold cross validation [5, Section 5.3] needs to be conducted during model training, the training data will be split into ten partitions based on patient IDs so that all healthcare visits for bronchiolitis of the same patient will be put into the same partition.

*Data pre*-*processing* We will use standard techniques, such as imputation, to handle missing values and detect and correct/remove invalid values [1, 36]. For clinical and administrative attributes, we will use grouper models such as the Diagnostic Cost Groups (DCG) system to group procedures, diseases, and drugs and reduce attributes [116, Chapter 5].

*Input variables* Predictors of asthma diagnoses in bronchiolitis patients have not been fully identified. In our recent papers [80, 117], we compiled an extensive list of known predictors of asthma diagnoses in bronchiolitis patients. All known predictors stored in the Intermountain Healthcare enterprise data warehouse will be used as input variables. In addition, our data set contains attributes beyond the known predictors.

*Predictive models* As one predictive model does not fit all [118], separate predictive models will be developed for children presenting with bronchiolitis at <6, 6–12, and 13–24 months of age [119]. The final model will be the combination of all models. We will use the standard performance metric of the AUC [5].

##### User feedback

In either round after model building is completed, we will survey the five healthcare researchers using a combination of semi-structured and open-ended questions. We will gather quantitative outcome measures including prediction accuracy, time on task, satisfaction, self-efficacy for building machine learning predictive models with big clinical data, adequacy, trustworthiness, and documentation quality as described in Table 1. The questionnaire will include a text field for user comments on MLBCD, if any. We will incorporate suggestions from these comments and refine/finalize MLBCD.

**Table 1:** Description of the dependent variables

Variable	Description
Prediction accuracy	AUC achieved by the predictive model built
Time	Number of hours spent on building the predictive model
Satisfaction	Responses to three questions: (1) How satisfied were you with the predictive model built? (2) How easy was the predictive model building process? and (3) How much effort did it take to complete the predictive modeling task? Ratings are on a 1–7 scale with anchors of not at all/completely; difficult/easy; and a lot of effort/little effort
Self-efficacy for building machine learning predictive models with big clinical data	Response to the question: overall how confident are you about your ability to build machine learning predictive models with big clinical data [129]? Rating is on a 1–5 scale with anchors of not at all/completely confident
Adequacy	How sufficiently do you think MLBCD supports building machine learning predictive models with big clinical data? Rating is on a 1–7 scale with anchors of not at all/sufficiently
Trustworthiness	How much sense do you think the predictive models make clinically? Rating is on a 1–7 scale with anchors of not at all/completely.
Documentation quality	Responses to two questions: (1) How comprehensive is MLBCD’s user manual? (2) How easy is MLBCD’s user manual to understand? Ratings are on a 1–7 scale with anchors of not at all/comprehensive; and difficult/easy

A formal user satisfaction survey will be conducted using the System Usability Scale (SUS), a publicly available 10-item scale [120, 121]. The scale provides an overall satisfaction rating for products. Higher scores indicate more positive usability perceptions [122]. The SUS is a widely used industry standard. A meta-analysis [123] endorsed the SUS above other instruments, as it applies to various products, is easy to use, and has a score that is easy to interpret. The scale has acceptable psychometrics. The internal consistency reliability ratings using Cronbach’s alpha ranged from 0.85 to 0.91 [123]. Factor analysis revealed one factor: usability [123]. The scale correlates well with other usability questionnaires for adequate concurrent validity [122].

*Analysis* We will conduct a qualitative analysis using the accepted inductive approach recommended by Patton et al. [94, 124]. Textual comments provided by the five healthcare researchers will be loaded into ATLAS qualitative analysis software [125]. We will highlight quotations and text relevant to the issue of using MLBCD. Quotations will be reviewed, categorized into pre-codes, and aggregated into categories after several iterations. General themes will be identified by synthesis of categories.

Quantitative analyses will consist of summing the scores on the SUS and reporting descriptive statistics on each quantitative outcome measure.

#### Stage 3: Test MLBCD’s utility

Using the same test case in “Stage 2: Evaluate MLBCD’s usability among healthcare researchers”, we will evaluate MLBCD’s utility in two parts. Part 1 compares healthcare researchers with MLBCD to computer scientists without MLBCD representing the state of the art of model building. Part 2 compares computer scientists with and without MLBCD.

##### Subject recruitment

We will recruit volunteer healthcare researchers using the same method described in “Stage 1: Assess user needs, preferences, and requirements”. Through personal contact and announcement in our institute’s email lists and course lectures, volunteer computer scientists among graduate students, staff, and faculty with machine learning background will be recruited at the University of Utah. All test participants will be current on information security and privacy policy training approved by the University of Utah.

In part 1, we will recruit 25 healthcare researchers who are involved in neither the Stage 1 nor the Stage 2 study, have limited computing expertise, and are familiar with pediatric asthma and bronchiolitis. After providing consent, each will be given a copy of MLBCD’s user manual and the metadata document (see “Subject recruitment”). In addition, we will recruit 25 computer scientists. After providing consent, each will be given a copy of the metadata document. They will manually tune machine learning models and spend more time on the study than the other test participants.

In part 2, we will recruit 25 computer scientists not involved in part 1. After providing consent, each will be given a copy of MLBCD’s user manual and the metadata document. The metadata document describes each attribute in the data set in detail. If any computer scientist needs clinical input such as explaining clinical concepts during the study, we will arrange a clinician to provide consultation.

##### Build predictive models

In part 1, each of the 25 healthcare researchers will build predictive models with MLBCD. Each of the 25 computer scientists will build models without MLBCD. In part 2, each of the 25 computer scientists will build models with MLBCD. Finally, we will select from all of these models the one achieving the highest AUC, use MLBCD to refine it if possible, and determine our final model.

*Model comparison and sample size justification* In part 1, we will compare the AUCs achieved by the 25 healthcare researchers with MLBCD to those achieved by the 25 computer scientists without MLBCD. We will use two one-sided equivalence tests [126] to test our primary hypothesis that healthcare researchers can use MLBCD to achieve similar prediction accuracy as computer scientists without MLBCD. Here as an approximation, we treat AUCs from different test participants as independent measures by regarding participants as a random sample from the population. A sample size of 25 instances per group will achieve 80 % power at a significance level of 0.05 when the true standardized difference of AUC between the two groups is 1.29 and the equivalence limits of the standardized difference are −2 and 2. We would regard the Stage 3 study successful if the non-equivalence hypothesis is rejected.

In part 2, we will compare the AUCs achieved by the 25 computer scientists without MLBCD to those achieved by the other 25 computer scientists with MLBCD. We will use a one-sided independent-sample *t* test to test the secondary hypothesis that computer scientists with MLBCD can achieve higher prediction accuracy than those without MLBCD. A sample size of 25 instances per group will have 80 % power at a significance level of 0.05 to detect a standardized difference of AUC between the two groups of 0.7.

We will record and describe the number of hours each test participant spent building the predictive model.

If our models cannot achieve high prediction accuracy, we will develop separate models for different subgroups of bronchiolitis patients defined by characteristics such as prematurity, co-morbidity, or type of healthcare visit for bronchiolitis. If both healthcare researchers and computer scientists still achieve low prediction accuracy, e.g., because asthma diagnoses are not predictable, we cannot tell whether MLBCD is effective. In this case, we will choose another test case, where it is known that some machine learning algorithm can achieve high prediction accuracy and statistical methods cannot. Statistical methods are known to perform poorly for predicting asthma diagnoses in children [80].

#### Ethics approval

We have already obtained institutional review board approvals from the University of Utah and Intermountain Healthcare for the study on evaluating MLBCD.

#### Preliminary user study

In preparation for the formal evaluation of MLBCD, we conducted a preliminary user study to assess user needs. We recruited two volunteer healthcare researchers with limited computing expertise from the University of Utah Health Sciences Center. Both of them were given a metadata document detailing tables and columns containing attributes to be used for the evaluation study. We first asked the two healthcare researchers to imagine building machine learning predictive models for the test case described in “Stage 2: Evaluate MLBCD’s usability among healthcare researchers” using existing software such as an Oracle database and Weka [5]. Both of them mentioned that without asking for help from computing professionals, they did not know how to transform big EAV data into relational table formats, such as performing pivot operations by writing complex SQL queries. One of them knew how to perform pivot operations in Excel, which works for only small data sets. Neither of them knew how to choose an appropriate machine learning algorithm and hyper-parameter values. In fact, one of them had never heard of hyper-parameters before and did not know that hyper-parameter values could be chosen in machine learning software such as Weka. Next, we described to the two healthcare researchers at a high level how MLBCD will work and showed them an early-stage prototype graphical user interface for a basic pivot function: pivoting on a single EAV table to generate a relational table. Both of them mentioned that a software tool like MLBCD would be very useful to them and greatly reduce the barriers for them to build machine learning predictive models by themselves. Also, the prototype graphical user interface for the basic pivot function is intuitive for them to understand. Findings from the preliminary user study confirmed the need for a user-friendly software tool and supported conducting a formal evaluation described in “Stage 1: Assess user needs, preferences, and requirements**”**.

### Related work

As described in our review paper [12], computer science researchers have developed multiple automatic selection methods for machine learning algorithms and/or hyper-parameter values. Most of these methods focus on either searching for an effective algorithm or searching for an effective combination of hyper-parameter values. Only a few methods can select both algorithms and hyper-parameter values simultaneously. None of these methods can efficiently handle big clinical data in the presence of a large variety of algorithms, limiting these methods’ usefulness in practice. The automatic selection method described in this paper addresses the limitations of these methods. Table 2 shows a summary of the comparison between the automatic selection method used in MLBCD and existing automatic selection methods for machine learning algorithms and/or hyper-parameter values. A detailed review of existing methods is provided in our paper [12].

**Table 2:** MLBCD vs. existing automatic selection methods for machine learning algorithms and/or hyper-parameter values

Method	Select algorithms	Select hyper-parameter values	Can efficiently handle big data	Can handle a wide range of algorithms	Can handle various types of hyper-parameters
MLBCD	✓	✓	✓	✓	✓
[130]	✓	×	×	×	×
[57, 131]	✓	×	×	✓	×
[9, 51, 54, 55, 132]	✓	×	✓	✓	×
[58, 61]	×	✓	×	×	✓
[10, 59, 133–138]	×	✓	×	×	×
[7, 8, 13, 14]	✓	✓	×	✓	✓

Google provides the Google Prediction API [127] that has some degree of automation for machine learning problems. The API’s internal workings have never been published. Also, the API puts a limit of ≤2.5 GB on the training data size. Amazon provides a service for machine learning: Amazon Machine Learning [128]. This service uses only two machine learning algorithms: logistic regression and linear regression. For many predictive modeling problems, other algorithms significantly outperform these two algorithms. Due to privacy concerns, many healthcare systems allow researchers to use their clinical data only behind the firewall. Consequently, machine learning services hosted by external companies become essentially inaccessible to researchers in these healthcare systems. In comparison, MLBCD can be installed on computers behind the firewall and are accessible to researchers in any healthcare system.

## Conclusions

We describe the design of MLBCD, a new software system aiming to enable healthcare researchers with limited computing expertise to develop machine learning predictive models. MLBCD supports the whole process of iterative machine learning on big clinical data, from clinical parameter extraction to model building and evaluation. MLBCD will open the use of big clinical data to many healthcare researchers and increase the ability to foster biomedical discovery and improve care. We are currently in the process of building MLBCD.

## Abbreviations

AUC: area under the receiver operating characteristic curve
CSV: comma-separated values
EAV: Entity–Attribute–Value
EMR: electronic medical record
i2b2: Informatics for Integrating Biology and the Bedside
JDBC: Java Database Connectivity
MLBCD: machine learning for big clinical data
OMOP: observational medical outcomes partnership
PCORnet: the National Patient-Centered Clinical Research Network
ROC: receiver operating characteristic
SQL: structured query language
